# Supplementary material for: Cyclic di-GMP Modulates a Metabolic Flux for Carbon Utilization in Salmonella enterica Serovar Typhimurium
Source: Microbiol Spectr. 2023 Feb 6;11(2):e03685-22. doi: 10.1128/spectrum.03685-22 (PMC10100716; doi:10.1128/spectrum.03685-22)
Supplement: Supplemental file 1 — Tables S1 and S2 and Fig. S1 to S5. Download spectrum.03685-22-s0001.pdf, PDF file, 0.6 MB [file spectrum.03685-22-s0001.pdf]

Table S1. Primers used in strain and plasmid construction

Primer	Sequence (5′ to 3′)
sgrS-RF	GCA ATT TTA TTA TCC CTA TAT TAG GCC AAT AAT ATC TTT CGT GTA GGC TGG AGC TGC TTC
sgrS-RR	AAA CGC CGT CCC ATC ATC GTC ATC CAG AAA CAG CCG AAA TAT TCC GGG GAT CCG TCG ACC
pAdrA-CF	ATA AGA TCT GGT AGG AGG TTT CTA AAT GTT CCC AAA
pAdrA-CR	AAA CTC GAG TTA GGA AAA ATC AGA GGC GCT CA
pCra-CF	AAT ATA GGA TCC TAA CGA TTT TAA CCC ATG
pCra-CR	TTTAAA GTC GAC TGA TAC GCA TTT GTT TGA
Cra-6His-F	AAATTT GAGCTC AGGAGG AATTCA CC ATGA AAC TGG ATG AAA TCG CTC GGC TGG CCG GTG TCT
Cra-6His-R	TTT TCT AGA TTA GTG ATG GTG ATG GTG ATG GCT ACG GCT CAG AAT GCC GCG ACG ATA

Table S2. Primers used in RT-qPCR and EMSA

Primer	Sequence (5′ to 3′)
rpoD-RT-F	AATGCTCCGTTGCCGAATAC
rpoD-RT-R	ATCGACAAAGCCGGTGATAAGA
adrA-RT-F	GTGCATGAACGGCTAAATACCTT
adrA-RT-R	AATGACCGATTTGTGGCGTTA
Cra-RT-F	ACG TTC CAT TGG TCT GGT GAT
Cra-RT-R	TTC AGA ACA GGC GAT CAG CA
fruB-RT-F	CCG GAA ATA AAG AAG AGG CCA
fruB-RT-R	GAA AGG TCG AGG TTT GCT GTT
fruK-RT-F	AGT GAA CCT GGT GAA AAC CAC
fruK-RT-R	ATC TTT ACC GAG AAA ACC GCC
fruA-RT-F	CGA CAA TCC GAA TGA TGC TGA
fruA-RT-R	ACC AAT ATC CCC CAG CCA AA
ptsG-RT-F	TGCAATCGCAGCGTATATGTTT
ptsG-RT-R	CAGCACCACACCGGTAAAAA
ptsH-RT-F	TGTGACTTCCAACGGCAAAA
ptsH-RT-R	ACCGCTTTCTGCTCATCTTCA
ptsI-RT-F	GGTGAAAAACGGCGATTATCTG
ptsI-RT-R	AGTTTAGCCAGTTCCGCTTTCTC
manX-RT-F	TTAGGCGAGCAGGAAAACGT
manX-RT-R	CTGGTATCGAGTTTAGCCAACTGA
rmanY-RT-F	CTGCACGTTTCTTCTCTGTTCCT
manY-RT-R	CGCATTCAGCATTCCCTGTA
manZ-RT-F	AGAATAATGACGCGCGTAAACA
manZ-RT-R	GCACGCTTTTCTTCCATTGC
rocR-RT-F	AAGGTCCATTCGGTGATCCT
rocR-RT-R	AGGTCGCCGAGAAAATTGAG
sgrS-RT-F	CGCCAGTTCTGGTTGAGATATTT
sgrS-RT-R	TTCCGCGAGCATTTTTAAGC
crp-RT-F	CAGGGTGAAAAAGCAGAAACG
crp-RT-R	GGCCCAGTTCACCAATAAAATC
fis-RT-F	CGGTTAAACAGGCACTGAAGAA
fis-RT-R	ACGGGTGTATTGCATCACCAT
mtfA-RT-F	GCCCTGGAAAGCACAAGAAA

mtfA-RT-R	AACGTTCGGCTAGCGCAATA
cyaA-RT-F	TCGTTTGGACTCGATCCCTACT
cyaA-RT-R	TAAAAGCAGCGGCGGACTAA
soxS-RT-F	TGTCGCATCAGCAGATAATTCA
soxS-RT-R	GGAGTAGCCCGATTTTTTTGC
mlc-RT-F	TGGCGAAATTTTCCTTGCA
mlc-RT-R	AAAACCGATCAACGTGCGTAA
arcA-RT-F	GAAGCGGAAGGCTATGATGTATTC
arcA-RT-R	TCCCTGGCAGATTGATATCCA
fbp-RT-F	AGA AAC TCG ATC TGT TCG CGA
fbp-RT-R	GTG TTC GCA GCC TTC AAA GA
pps-RT-F	TGA ATG ATG TAG ACA GAG TTG G
pps-RT-R	CAT CGG CAG TGG TTG CAA AA
pck-RT-F	ATC CTG GTC TGG AAG GCT AT
pck-RT-R	CGT CGC GAA CAA TAT ACT TAT C
ppc-RT-F	AAC ACA TTC TTG ATC GCG TAG A
ppc-RT-R	CAG CAG CTC GTC ATT AGA CAA
pfkA-RT-F	GAT CGT ATG GTT CAG CTT GAC
pfkA-RT-R	AAT ATT TTC GTC ACG GAA TTC CG
pfkB-RT-F	ACA TCT TGT CGC ACT ACT GG
pfkB-RT-R	AAA CGA TAC TGT TCG CCG CT
pykA-RT-F	ACC GCG ATA ACA ACC TTG AGA
pykA-RT-R	TCT CAC GGA CTT TAT CAG CAC
pykF-RT-F	ACG TGA TGC GTC TGA ACT TCT
pykF-RT-R	AGT ATC AAG CAG AAT AGC GGC
glmS-RT-F	GCT GAA ATC CTT CTC GAA GGT
glmS-RT-R	AGC ATC TGG ACT TTA CCG AG
glmU-RT-F	CCG ATA TTC CTA AAG TGC TGC
glmU-RT-R	TTT AAC AAC TCG CCA CCG TG
fruB-EMSA-F	GCTGCCCGTCAGAAAATAAAC
fruB-EMSA-R	ATGTCCTGAACCGATAACTGG
ptsG-EMSA-F	TGTTCCATATTGACGCGTCTC
ptsG-EMSA-R	AGCATCAGCGATTTACCGAC
manX-EMSA-F	AGACAACTGGTTTTTGGCAAAG
manX-EMSA-R	GCATTTCGGCTGTTTTAAGTAAC

Supplementary Figure 1

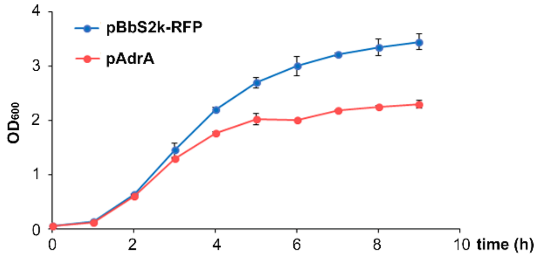

FIG S1 Bacterial growth curves were plotted when c-di-GMP was overexpressed. Wild-type *Salmonella enterica* serovar Typhimurium was transformed with pAdrA or its empty plasmid, pBbS2k-RFP and incubated in Luria-Bertani (LB) broth containing 10 ng/ml anhydrotetracycline (aTc) for *adrA* induction. Bacterial growth was measured at 600 nm every hour.

Supplementary Figure 2

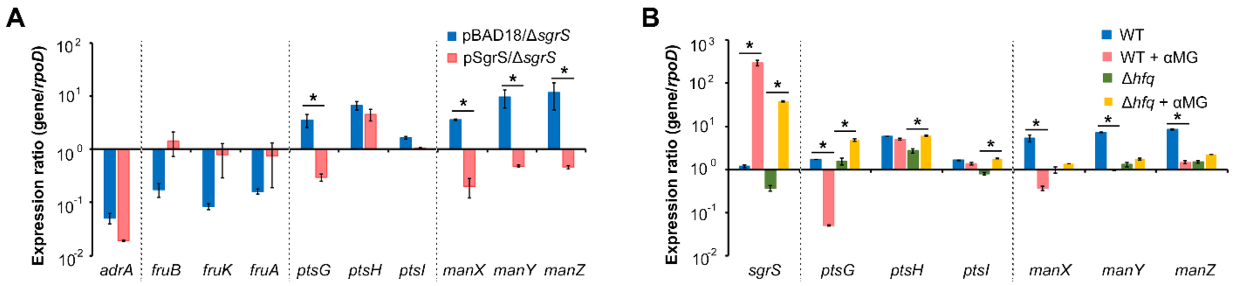

FIG S2 SgrS decreased mRNA levels of PTS genes with the aid of Hfq. (A) *S.* Typhimurium Δ*sgrS* mutant strains containing pSgrS and its empty plasmid, pBAD18, were cultivated in M63 minimal medium broth supplemented with 0.2% arabinose for 7 h. (B) *S.* Typhimurium WT and Δ*hfq* strains were cultivated in LB broth for 2 h and subsequently treated with 0.5% methyl α-D-glucopyranoside (αMG) for an additional 3 h. The levels of mRNA were relatively measured through RT-qPCR using C_t values of *rpoD* as the reference. Statistical significance is indicated with *****, *P* value < 0.05.

Supplementary Figure 3

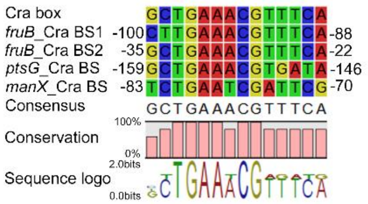

FIG S3 The consensus Cra boxes were predicted at sequences upstream of *fruB*, *ptsG*, and *manX*. Putative Cra binding sequences were aligned using CLC Sequence Viewer 7.6.1 software. Two Cra boxes were predicted upstream of *fruB* (*fruB*_Cra BS1 and BS2), while one Cra box each was anticipated upstream of *ptsG* (*ptsG*_Cra BS) and *manX* (*manX*_Cra BS). Numbers next to the highlighted sequences indicate the distance from the start codon of each gene. The sequence logo indicates the frequency of bases at each position.

Supplementary Figure 4

DNA (40 ng)	+	+	+	+	+	+	+	+	+	+
Cra (16 μM)	-	+	+	+	+	-	+	+	+	+
c-di-GMP (250 μM)	-	-	-	-	-	+	+	+	+	+
FBP (mM)	-	1	5	10	25	-	1	5	10	25

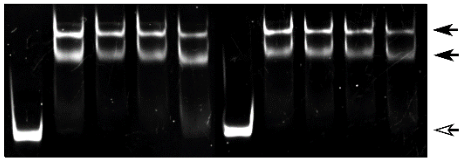

DNA (40 ng)	+	+	+	+	+	+	+	+	+	+
Cra (4 μM)	-	+	+	+	+	-	+	+	+	+
c-di-GMP (250 μM)	-	-	-	-	-	+	+	+	+	+
FBP (mM)	-	1	5	10	25	-	1	5	10	25

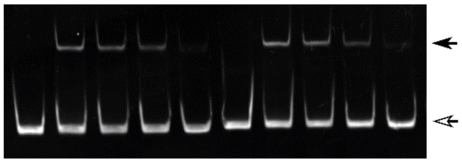

DNA (40 ng)	+	+	+	+	+	+	+	+
Cra (8 μM)	-	+	+	+	-	+	+	+
c-di-GMP (250 μM)	-	-	-	-	+	+	+	+
FBP (mM)	-	1	5	10	-	1	5	10

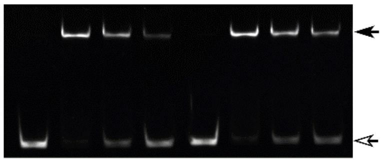

FIG S4 Cyclic-di-GMP tended to facilitate Cra to bind to P_{fruB} and P_{manX}. Cra-His_6 at different concentrations (4, 8, and 16 μM) was incubated with three different DNA fragments containing promoters of *fruBKA* (P_{fruB}), *ptsG* (P_{ptsG}), and *manXYZ* (P_{manX}), in the presence or absence of c-di-GMP (250 μM). Fructose-1,6-bisphosphate (FBP; 0 to 25 mM) was added to inhibit Cra from binding to the promoters. The complexes between Cra-His_6 and DNAs were analyzed using 6% native polyacrylamide gel. Black arrows indicate the DNA-protein complexes, while white arrows are free DNAs of P_{fruB} (237 bp), P_{ptsG} (388 bp), and P_{manX} (331 bp), respectively.

Supplementary Figure 5

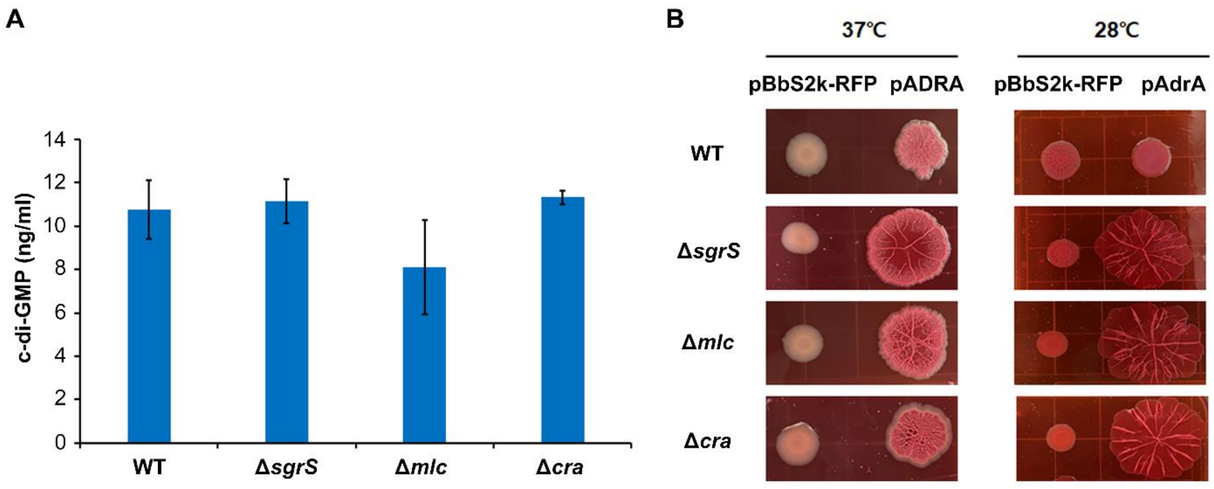

FIG S5 The catalytic activity of AdrA was not altered by the absence of SgrS, Mln, or Cra. (A) *Salmonella* strains including wild-type and three mutants (Δ*sgrS*, Δ*mlc*, and Δ*cra*) were transformed with pAdrA and cultivated in LB broth containing 10 ng/ml aTc for *adrA* induction. Cyclic-di-GMP concentrations were measured at 18 h. (B) *Salmonella* strains were transformed with pAdrA or its empty plasmid pBbS2k-RFP and spiked onto NaCl-depleted LB agar plates containing Congo red (40 μg/ml) and aTc (20 ng/ml). The plates were incubated at 28 and 37℃.
